# Test of a Retinal Nerve Fiber Bundle Trajectory Model Using Eyes With Glaucomatous Optic Neuropathy

**DOI:** 10.1167/tvst.11.7.7

**Published:** 2022-07-12

**Authors:** Zane Zenon Zemborain, Emmanouil Tsamis, Sol La Bruna, Ari Leshno, Carlos Gustavo De Moraes, Donald Charles Hood

**Affiliations:** 1 Department of Psychology, Columbia University, Schermerhorn Hall, New York, NY, USA; 2 Department of Biomedical Engineering, Duke University, Durham, NC, USA,; 3 Bernard and Shirlee Brown Glaucoma Research Laboratory, Edward S. Harkness Eye Institute, Department of Ophthalmology, Columbia University Medical Center, New York, NY, USA; 4 Sackler Faculty of Medicine, Tel Aviv University, Tel Aviv, Israel

**Keywords:** Glaucoma, Optical Coherence Tomography, Arcuate

## Abstract

**Purpose:**

To test a model of retinal nerve fiber bundle trajectories that predicts the arcuate-shaped patterns seen on optical coherence tomography (OCT) retinal nerve fiber layer (RNFL) probability/deviation maps (p-maps) in glaucomatous eyes.

**Methods:**

Thirty-one glaucomatous eyes from a database of 250 eyes had clear arcuate-shaped patterns on RNFL p-maps derived from an OCT cube scan. The borders of the arcuate patterns were extracted from the RNFL p-maps. Next, the trajectories from an arcuate model were compared against these borders via a normalized root-mean-square difference analysis. The model's parameter, β, was varied, and the best-fitting, initial clock-hour position of the trajectory to the border was found for each β. Finally, the regions, as determined by the arcuate border's best-fit, initial clock-hour positions, were compared against the abnormal regions on the circumpapillary retinal nerve fiber layer (cpRNFL) profile.

**Results:**

The arcuate model's mean β_Sup_ and β_Inf_ parameters minimized large differences between the trajectories and the arcuate borders on the RNFL p-maps. Furthermore, on average, 68% of the cpRNFL regions defined by the arcuate border's best-fit, initial clock-hour positions were abnormal (i.e., below the ≤5% threshold).

**Conclusions:**

The arcuate model performed well in predicting the borders of arcuate patterns seen on RNFL p-maps. It also predicted the associated abnormal regions of the cpRNFL thickness plots.

**Translational Relevance:**

This model should prove useful in helping clinicians understand topographical comparisons among different OCT representations and should improve structure-structure, as well as structure-function agreement analyses.

## Introduction

Glaucoma is a progressive optic neuropathy in which the selective loss of retinal ganglion cells (RGC) and their axons results in thinning of the retinal nerve fibers.^1^ It typically presents with a specific pattern of retinal nerve fiber loss and visual field (VF) defects, which is arcuate in shape. This pattern, unsurprisingly, corresponds to the trajectory of retinal nerve fiber bundles traveling to the optic disc.

Because this pattern is a necessary condition for glaucoma, we have argued for diagnostic methodologies that focus on arcuate-shaped topographical patterns, as well as structure-structure and structure-function agreement.^2^^–^^6^ In particular, we have developed and tested a one-page optical coherence tomography (OCT) report that allows for an examination of the topographic agreement among depictions of OCT information (e.g., circumpapillary retinal nerve fiber layer [cpRNFL], RNFL, and RGC maps).^2^^–^^5^ In addition, we have performed quantitative comparisons on the abnormal regions on the RNFL and RGC probability (p-) maps on this report to the abnormal regions of VF deviation maps. These quantitative comparisons outperformed more traditional metrics.^6^^,^^7^ Hence, the evaluation of structure (OCT)-structure (OCT) and structure (OCT)-function (VF) agreement is of paramount importance for the detection of glaucoma.^5^

However, although commercial versions of our report have made assessment of OCT-OCT and OCT-VF comparisons easier, they can be improved. First, it can be a challenge for clinicians to understand the topographical relationship among the different thickness plots, probability (p-) maps, and circle b-scan images within the current OCT reports. Second, the 24-2 VF covers a larger retinal region than the commercial OCT scans. For example, the nasal defect, considered an early VF sign of glaucomatous damage, falls outside the region scanned by commercial OCT instruments.

To address these problems, we have suggested using a model,^8^ proposed by Jansonius et al.,^9^^,^^10^ that predicts the path taken by RNFL bundles as they travel from the RGCs to the disc. In this study, we describe a modification of the Jansonius et al. model, which incorporates the predicted pathway into existing glaucoma reports. The overall goals of this research are to make it easier, in the future, to (a) see the relationship between abnormal regions on RNFL p-maps and the associated portions of the cpRNFL images and thickness plots and (b) pair abnormal 24-2 VF locations to the extended trajectories of abnormal regions on RNFL p- maps.

To explore the extent to which these goals could be met, we performed a validation of the arcuate model, a modified retinal nerve fiber bundle trajectory model, using eyes with glaucomatous optic neuropathy. We tested the hypothesis that the mean of the main parameter (β_Sup_ and β_Inf_) of the Jansonius et al.^9^^,^^10^ model would yield trajectories which would consistently align well to the edges of arcuate-shaped patterns in the RNFL p-maps.

## Patients and Methods

### Patients

Thirty-one glaucomatous eyes from 31 participants (mean age 67.9 years, range 40–84 years) came from a database of 250 glaucomatous or suspect eyes. These 31 were selected because of clear, arcuate-shaped patterns on RNFL p-maps as described below. There were 17 arcuate patterns in the superior retinas and 30 arcuate patterns in the inferior retinas. The eyes had average mean deviations (MD) of −2.11 ± 1.85 dB and −2.75 ± 2.49 dB for the 24-2 and 10-2 VFs, respectively. Additionally, all eyes had a spherical equivalent refraction between −6 and 6 diopters.

All participants were recruited as part of an observational, prospective, case-controlled study, the Macular Damage in Early Glaucoma and Progression Study (PI: C Gustavo De Moraes; ClinicalTrials.gov Identifier: NCT02547740). Furthermore, they were considered to have glaucoma based on an automated, objective structure-function agreement method, which compared abnormal deviation values from 24-2 and 10-2 VF tests to abnormal ganglion cell layer (GCL) and RNFL probability values on the widefield OCT scan.^6^^,^^7^ We have previously reported on the high diagnostic performance of this method,^6^ as well as its superiority against other commonly used summary metrics.^7^

The Institutional Review Board of Columbia University prospectively approved this retrospective, observational, cross-sectional study. The approval included the collection, deidentification, exporting and analysis of OCT scans and other ophthalmic-related records. It followed the tenets of the Declaration of Helsinki and the Health Insurance Portability and Accountability Act. Written informed consent was obtained from all patients.

### OCT

**Figure 1.**
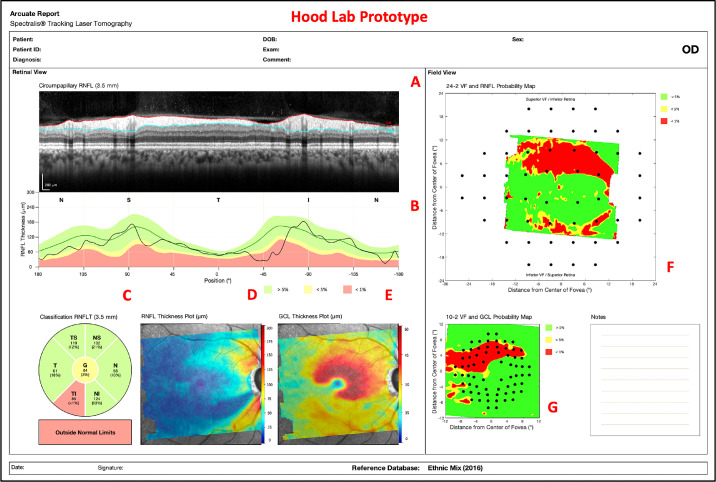
A custom, one-page Heidelberg Engineering (HE) report with circle b-scan (A), cp-RNFL plot (B), cpRNFL pie chart (C), RNFL thickness plot (D), GCL thickness plot (E), RNFL p-map (F), and GCL p-map (G).

All eyes were scanned with the Spectralis HRA+OCT with the Glaucoma Module Premium Edition protocol (Heidelberg Engineering, Heidelberg, Germany), which includes a single circle B-scan with a diameter of 3.5 mm and a horizontal volume scan (30° × 25°) with 61 horizontal b-scans, centered on the macula, and obtained along the fovea-to-disc (FD) center axis. Our one-page report, described in previous work,^3^^,^^4^ was constructed from the OCT scans (Fig. 1). It should be noted that this is similar to the commercial version available in some locations outside the United States.

### Arcuate Model: Modified Retinal Nerve Fiber Bundle Model

#### General Equation

The Jansonius et al. model^9^^,^^10^ describes the retinal nerve fiber bundle trajectories in a modified polar coordinate system, by the following:

(1) $$\begin{array}{c} \varphi \left(\varphi_{0},\;r)=\varphi_{0}+b(\varphi_{0}\right)\cdot {\left(r-r_{0}\right)}^{c(\varphi_{0})}\; \end{array}$$

For Equation 1, r_0_, r, φ_0_, φ, correspond to the initial radius (r_0_) around the center of the disc in a modified polar coordinate system, the radial component (r) of the polar coordinate system, the initial polar degree position (φ_0_) of the trajectory at its starting point, and the angular component (φ) of the polar coordinate system, respectively. The functions c(φ_0_) and b(φ_0_) are defined below in Equations 2 to 5.

### Auxiliary Equations

Parameter c determines the location of the curvature, whereas b determines the amount of curvature. Equations 2 to 5 define c and b for the superior-temporal region (60° ≤ φ_0_ ≤ 180°) and the inferior-temporal region (−180° < φ_0_ ≤ −60°). Jansonius et al.^9^^,^^10^ found that the parameters, β_Sup_ and β_Inf_, fell within ranges from −1.3 to −2.5 and 1.3 to 0.1, respectively.^10^ Additionally, they found that the mean β_Sup_ and β_Inf_ values were −1.90 and 0.70, respectively.

(2) $$\begin{array}{c} c_{\mathrm{Sup}}\left(\varphi_{0}\right)=1.9+1.4\;\tanh\left\{\left(\varphi_{0}-121\right)/14\right\}\; \end{array}$$

(3) $$\begin{array}{c} c_{\mathrm{Inf}}\left(\varphi_{0}\right)=1.0+0.5\;\tanh\left\{\left(-\varphi_{0}-90\right)/25\right\}\; \end{array}$$

(4) $$\begin{array}{c} b_{\mathrm{Sup}}\left(\varphi_{0}\right)\;=\exp\left(\beta_{\mathrm{Sup}}+3.9\;\tanh\left\{-\left(\varphi_{0}-121\right)/14\right\}\right)\; \end{array}$$

(5) $$\begin{array}{c} b_{\mathrm{Inf}}\left(\varphi_{0}\right)\;=-\exp\left(\beta_{\mathrm{Inf}}+1.5\;\tanh\left\{-\left(-\varphi_{0}-90\right)/25\right\}\right)\; \end{array}$$

### Modifications

**Figure 2.**
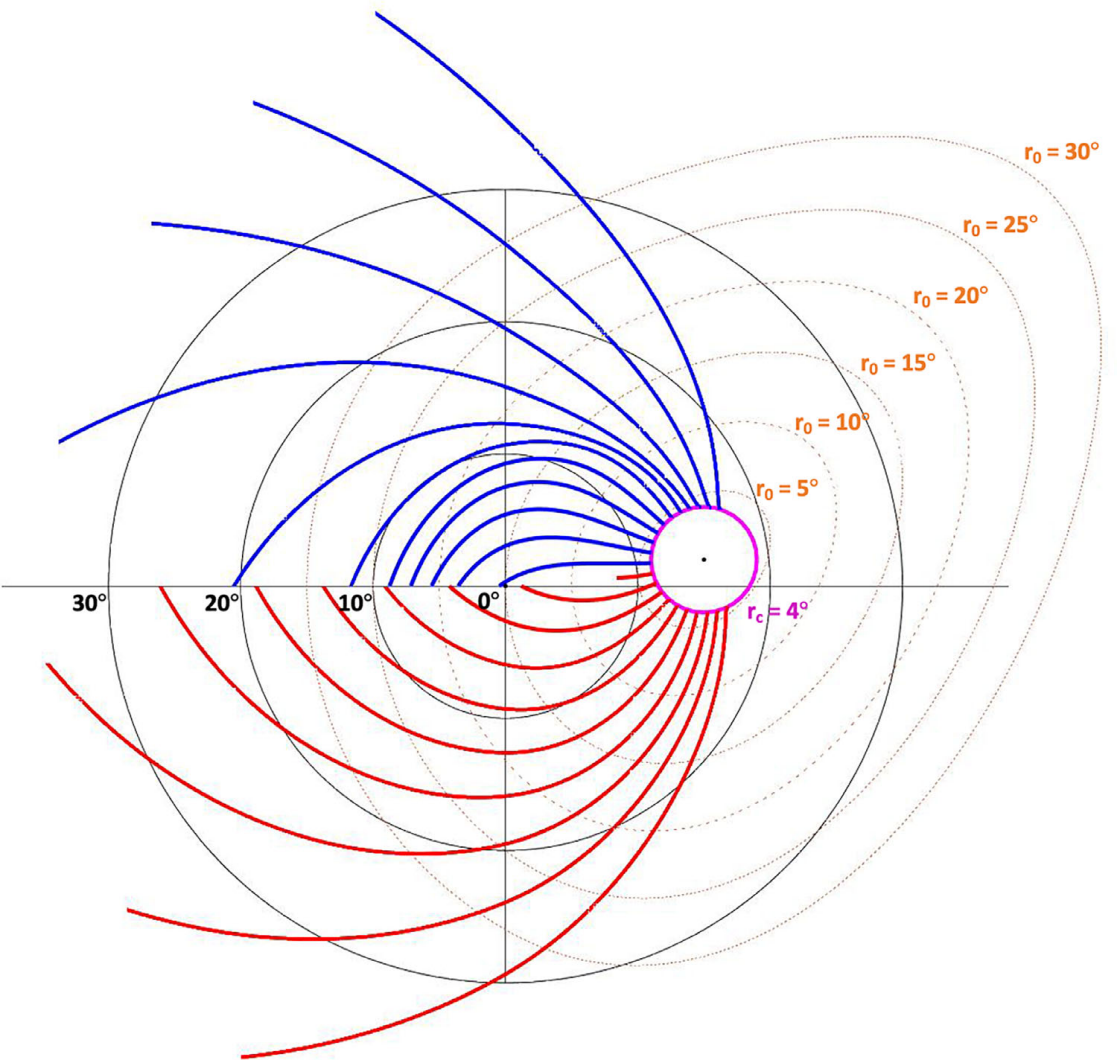
The superior (blue) and inferior (red) retinal nerve fiber bundle trajectories plotted in a cartesian coordinate system with its center at the fovea. Note: r_c_ varies with the circle b-scan radius.

A few modifications were made to the original model to apply it to the OCT p-maps: (1) Jansonius et al.^9^ placed the optic nerve head (ONH) center at an eccentricity of (15°, 2°) above the horizontal meridian in the cartesian coordinate system. We use (15°, 2°) to calculate the trajectories, but then re-center the trajectories to the position on the thickness plots and probability maps corresponding to the Bruch's membrane opening (BMO) center. (2) Jansonius et al. set r_0_ to a constant value of 4° in the modified polar coordinate system.^9^ We transformed the circle b-scan's degree radius (r_c_), acquired from the OCT imaging system, as well as the initial clock-hour positions (θ_c_), from the cartesian coordinate system to a set of paired r_0_ and φ_0_ values in the modified polar coordinate system. The trajectories were then calculated and transformed back to the cartesian coordinate system (Fig. 2). (3) Jansonius et al.^9^ assumed an FD angle of −7.59° for a right eye. We rotated the trajectories by the difference between −7.59° and the FD angle recorded by the OCT imaging system, or the “relative FD angle.” This should help correct for assessment variability (i.e., head tilt or eye rotation during acquisition). (4) Jansonius et al.^11^ noted that the raphe follows a horizontal line at the latitude of the fovea if the ONH center is assumed to be located at (15°, 2°). We also used the horizontal line at the latitude of the fovea as a boundary condition for the trajectories. It should be noted, however, that this boundary condition was rotated by the relative FD angle. (5) Jansonius et al.^10^ has an extended model for the nasal region of the retina. For simplicity, our nasal region (−60° < φ_0_ <60°) trajectories were approximated as lines perpendicular to the circle b-scan.

### Arcuate Border Extraction

**Figure 3.**
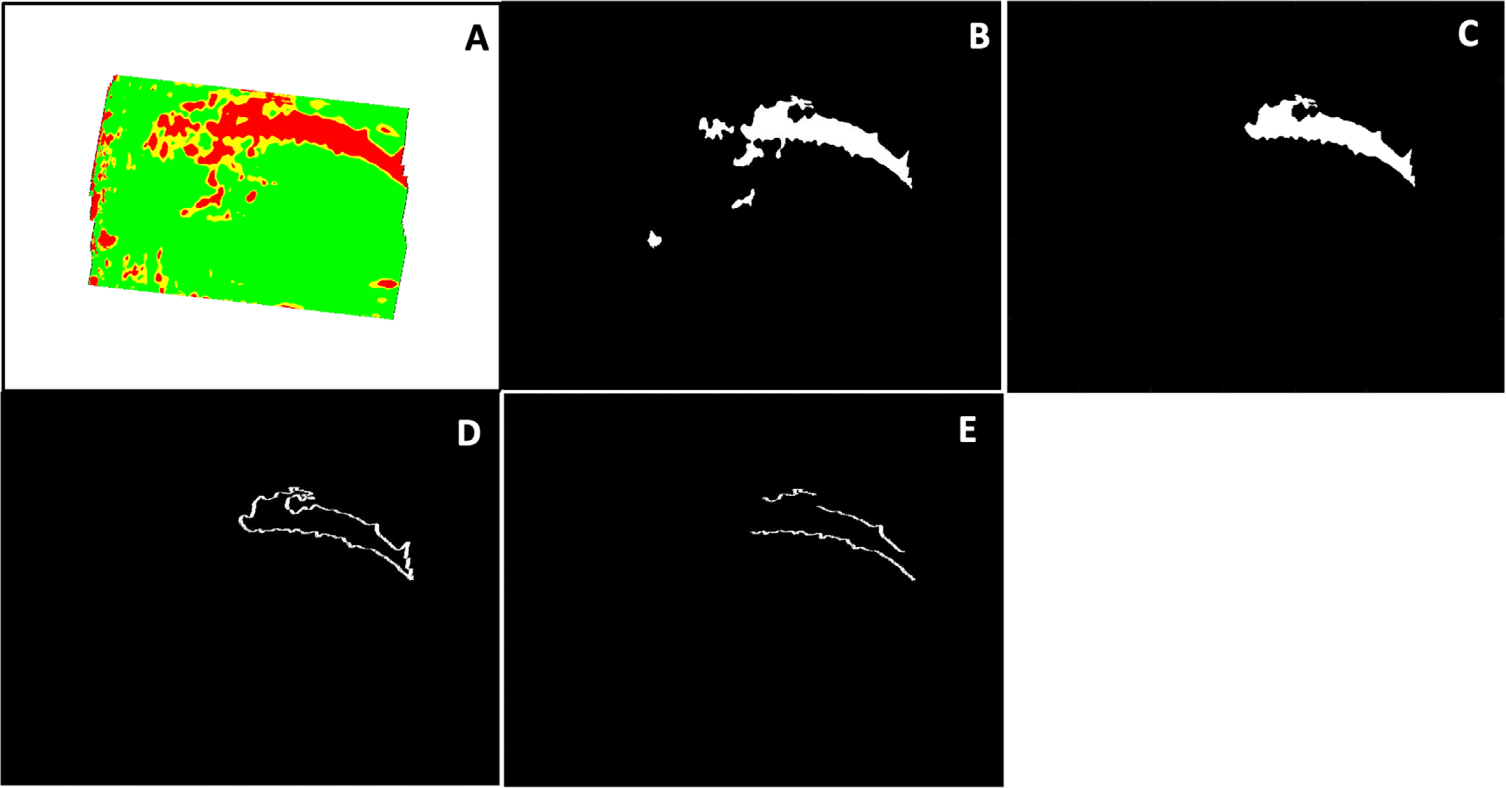
The arcuate border extraction was performed with the following steps: RNFL p-map (A), binary plot (B), arcuate-shaped pattern (ASP) (C), ASP perimeters (D), and arcuate borders (E).

Arcuate borders were extracted from the RNFL p-maps via the following steps: (1) We converted the RNFL p-map (Fig. 3A) to a binary plot (Fig. 3B) with >1% corresponding to a value of 0 and ≤1% corresponding to a value of 1. (2) We manually removed all non-arcuate-shaped abnormal regions (Fig. 3C). (3) We automatically removed everything other than the perimeter pixels from the binary plot (Fig. 3D). (4) We manually removed all non-arcuate regions of the arcuate borders (Fig. 3E).

### Root-Mean-Square Difference Analysis

The arcuate model required the following parameters: disc center, FD angle, fovea center, circle b-scan radius (r_c_), β value for superior or inferior retina, and initial clock-hour position (θ_c_). The disc center, FD angle, fovea center, and r_c_ were acquired from the OCT imaging system. Therefore, to fit the model for any given arcuate and individual, we need to estimate θ_c_ and β. However, it would not be practical in a clinical setting to get the optimal θ_c_ and β value for each arcuate border for each individual eye. A clinician should be able to approximate the optimal initial clock-hour position, θ_c_, by clicking on the arcuate pattern's border via our interactive arcuate report (described in a subsequent section). An optimal β value, however, would require iterating through all possible β values or acquiring more clinical information on spherical equivalent refraction, axial length, optic disc anatomy, and/or retinal blood vessel topography.^10^^,^^12^^,^^13^ For simplicity, we wanted to find the *single* best β_Sup_ and β_Inf_ value to use for all patients and all arcuate borders in their corresponding region of the retina. This was achieved by comparing the normalized root-mean-square (RMS) differences between the trajectories and the arcuate borders.

The arcuate model's trajectories were compared to the arcuate borders based on the following steps: first, points from the arcuate border and the trajectory with quantized radial distances from the ONH center were compared to one another. Specifically, we calculated the RMS difference between the arcuate border and the trajectory for each radial distance at a given initial, polar degree position (φ_0_). This step was performed for all 11 values within the ranges of the β_sup_ parameter for the model (or β_Inf_, if the arcuate was in the inferior retina). Then, for a given β value we found the minimum RMS difference among the various polar degree positions, which were iterated through via 0.25° steps. Subsequently, we normalized the RMS differences for the entire range ofβ values for a given arcuate; this was done by calculating their z-scores and converting them to percentiles. Note that we did not include any nasal positions as initial polar degree positions (φ_0_); that is, the φ_0_ ranged from 60° to 300°.

### Predicting the Abnormal Region on the cpRNFL Thickness Plot

**Figure 4.**
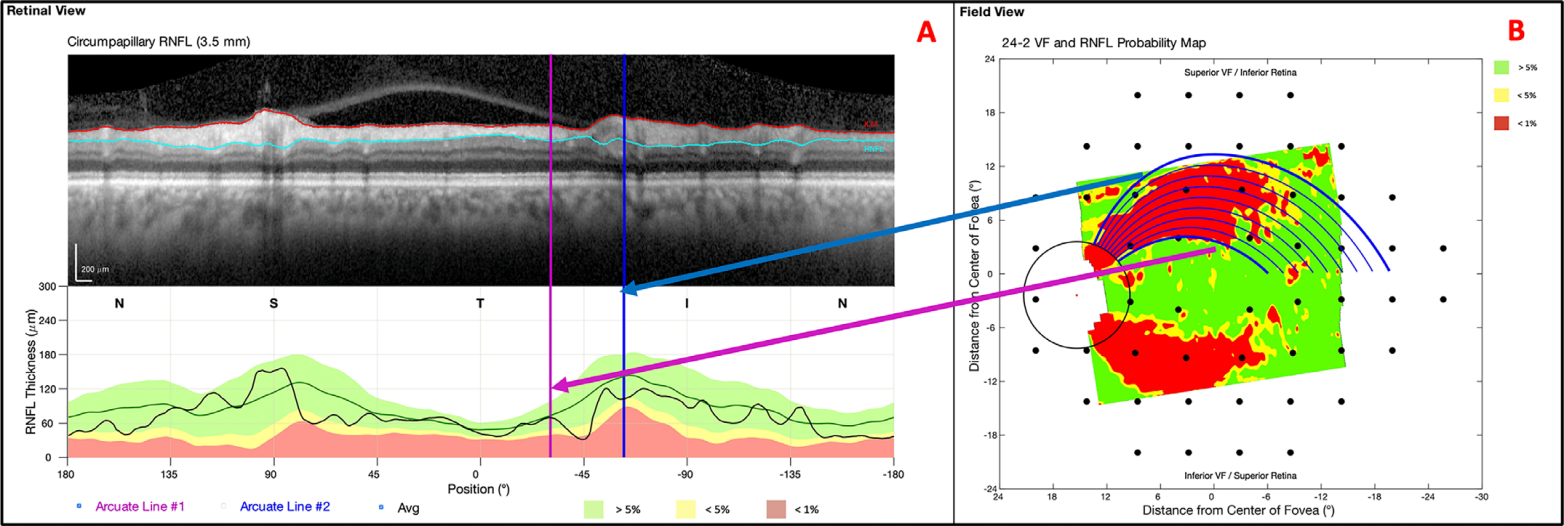
The arcuate borders on the cpRNFL plot (A) and on the RNFL p-map (B).

As a test of the model, we predicted the abnormal region on the cpRNFL thickness plot by setting the β_Sup_ and β_Inf_ parameters to the mean values, −1.90 and 0.70, based on the analysis in the preceding section. The cpRNFL regions, as determined by the RNFL p-map arcuate borders’ best-fit, initial clock-hour positions (Fig. 4B), were compared to abnormal regions on the cpRNFL plot (Fig. 4A). Note: the best-fit, initial, polar degree positions (φ_0_) were mapped to the best-fit, initial clock-hour positions (θ_c_) via a simple transformation.

The two best-fit, initial clock-hour positions were used as the bounds for the trajectory-determined regions on the cpRNFL plot. We then calculated the percentages of the cpRNFL regions that fell below ≤5% and ≤1% cpRNFL thresholds (yellow and red regions in Fig. 4A). Ultimately, the underlying assumption was that 100% of the trajectory-determined region would have abnormally thin cpRNFL (fall below ≤5% cpRNFL threshold) because RNFL damage typically appears at the disc before the temporal retina.^14^

### Arcuate Report

**Figure 5.**
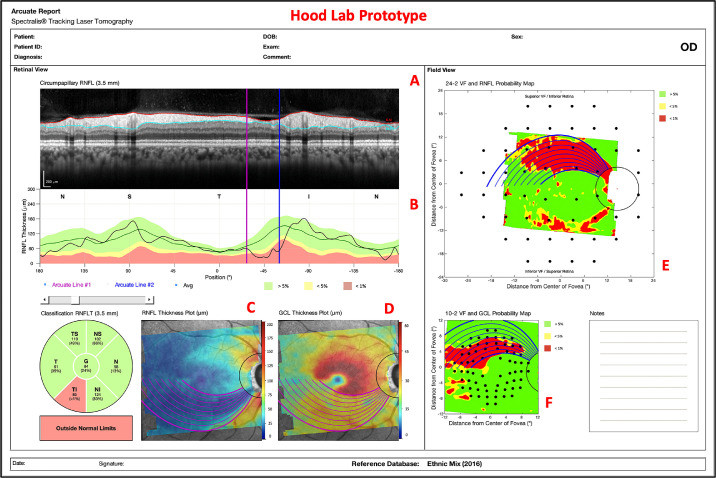
An arcuate report using HE scans with arcuate boundaries/lines on the circle b-scan (A), cpRNFL plot (B), RNFL thickness plot (C), GCL thickness plot (D), RNFL p-map (E), and GCL p-map (F).

The arcuate model was integrated into our pre-existing, one-page report in Figure 1 as shown in Figure 5. The arcuate boundaries/lines can be seen on the circle b-scan, RNFL thickness plot, GCL thickness plot, RNFL p-map, and GCL p-map. The arcuate lines on the thickness plots and p-maps initiate at the projected edge of the circle b-scan and terminate at either the FD line or the approximated raphe boundary. Additionally, numerous arcuate lines, within the bounds of a region-of-interest, are plotted to better capture the VF locations that they encompass. Finally, the arcuate report is interactive; a clinician can click on the various components of the report (e.g., cpRNFL plot or RNFL p-map) to reposition the arcuate boundaries/lines.

## Results

To be clinically useful, it is important that the arcuate model accurately and consistently predicts the trajectories of the arcuate patterns for all eyes. To achieve this, we performed a quantitative analysis of the arcuate model's performance under varied conditions and circumstances.

### Quantitative Analysis

#### RMS Difference Analysis

The model's parameter, β, was varied and the best-fitting, initial, polar degree position of the trajectory to the border was found for each β. To evaluate the performance of the various β values, we plotted their corresponding best-fitting, normalized RMS values onto a series of 95% confidence interval (CI) plots with each superior/inferior arcuate occupying a single point on each CI.

**Figure 6.**
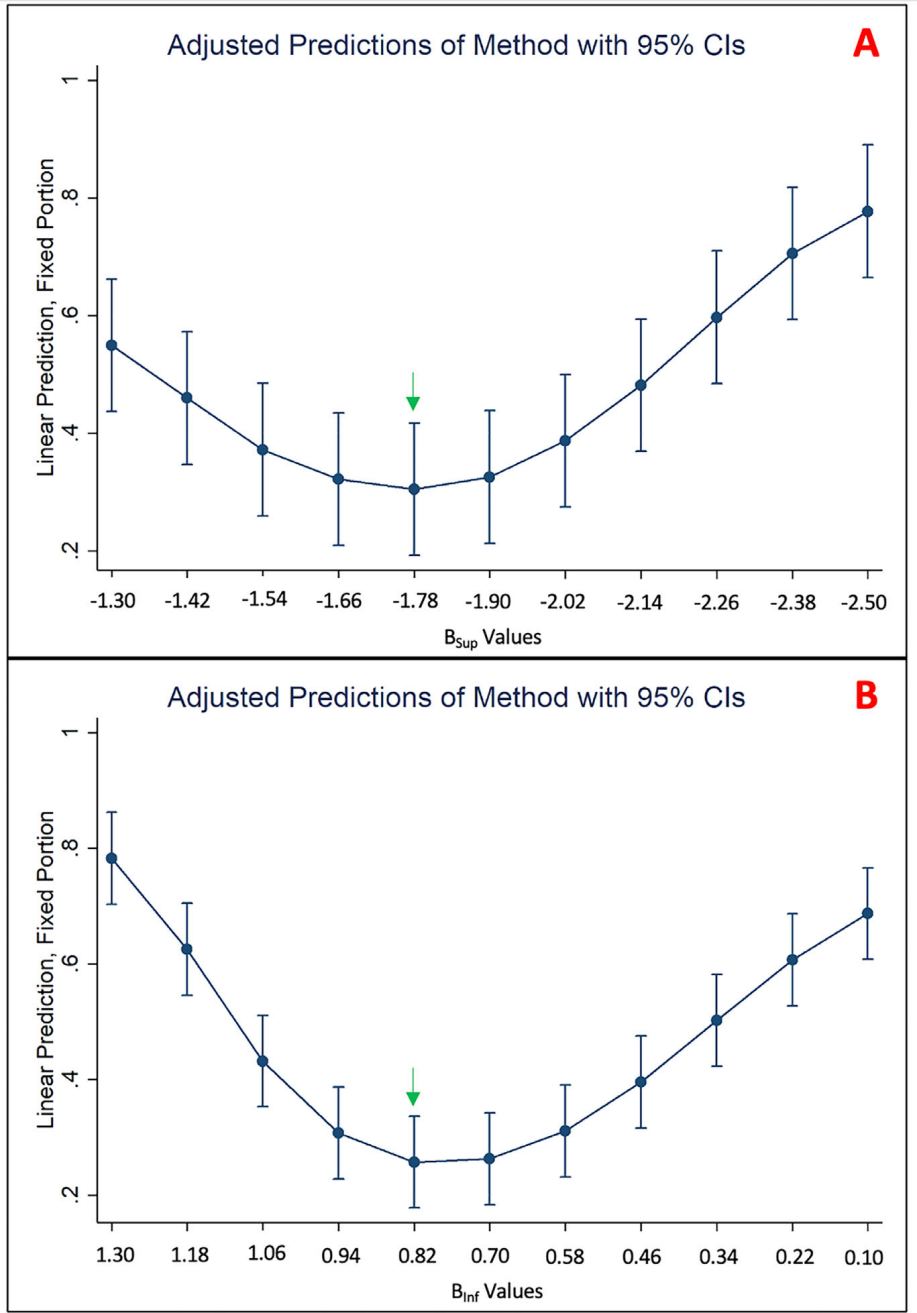
The 95% CIs of β_Sup_ performance (A) and β_Inf_ performance (B), as measured by normalized RMS difference, with the β values having the smallest 97.5th, 50th, and 2.5th percentiles marked (*green arrows*).

The CIs in Figure 6 show the normalized RMS difference values as a function of β_Sup_ (A) and β_Inf_ (B) values, which were each separated into 11 bins. Each CI contains the 97.5th percentile (upper dash), 50th percentile (dot), and 2.5th percentile (lower dash). The 97.5th, 50th, and 2.5th percentile values converge toward their minimum (Fig. 6; *green arrows*) slightly above the mean β_sup_ and β_Inf_ values. As such, we used these β_Sup_ and β_Inf_ values (−1.78 and 0.82, respectively) as the reference for our mixed effects linear model with analysis of margins. There was no statistically significant difference (*P* > 0.01) between the normalized RMS difference values produced by β_Sup_ values from −1.42 to −2.02. Similarly, there was no statistically significant difference (*P* > 0.01) between the normalized RMS difference values produced by β_Inf_ values from 0.94 to 0.58. The values outside of these ranges, however, had statistically significant differences from the references. As such, we can conclude that the mean β_Sup_ and β_Inf_ values, which fell within range of the reference values, produced smaller normalized RMS differences than the values at the extrema. Together, this information indicates that the mean β_sup_ and β_Inf_ values were unlikely to produce poor fits, relative to the other β values, between the trajectories and the arcuate borders.

It is not practical to obtain the optimal β values for an individual's eye. As such, we searched for the *single* β values that could fit the arcuate borders of *most* eyes. The mean β_Sup_ and β_Inf_ are logical choices and appear to consistently minimize poor fits.

#### Predicting the Abnormal Region on the cpRNFL Thickness Plot

**Figure 7.**
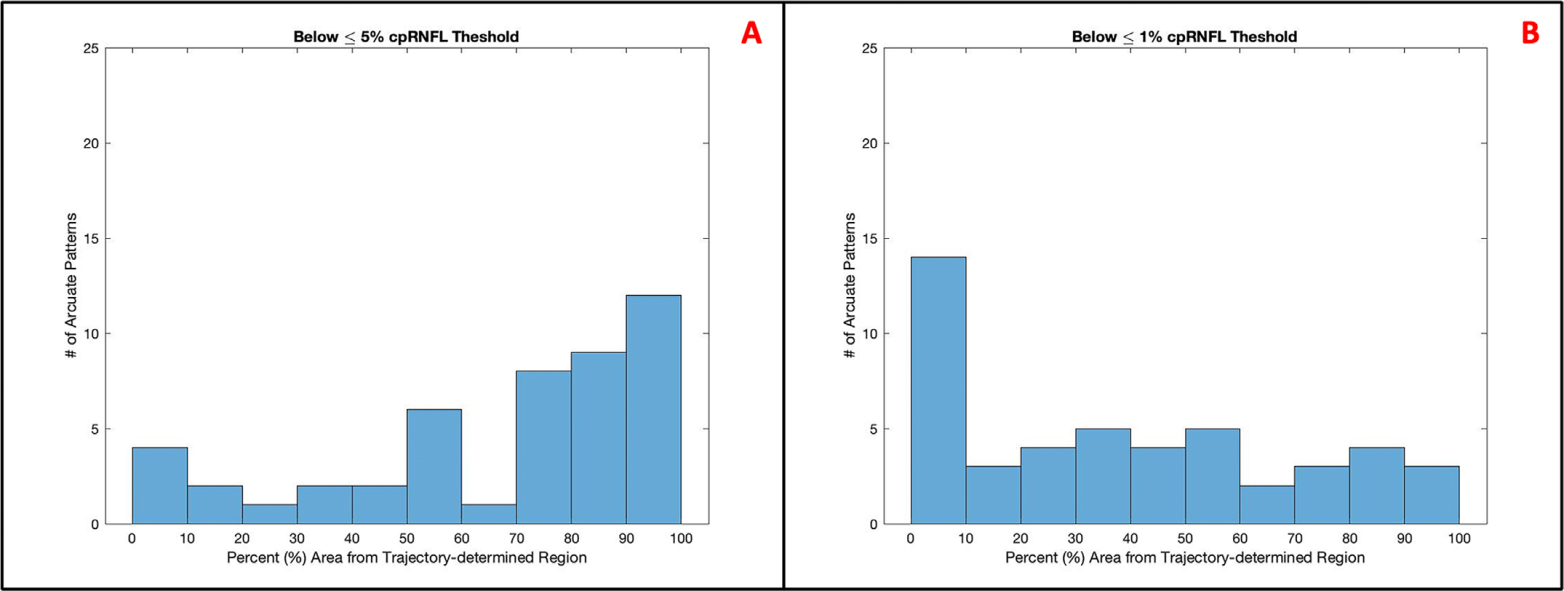
The histograms of the percent area from trajectory-determined regions that fell below the ≤5% (A) and ≤1% (B) cpRNFL thresholds.

The trajectory-determined regions on the cpRNFL plot for a given arcuate, on average, had 68% (standard deviation [SD]: 30%) and 37% (SD: 33%) of their area fall below the ≤5% and ≤1% cpRNFL thresholds, respectively. This indicates that the arcuate model, coupled with the arcuate borders, frequently predicted abnormal regions on the cpRNFL, as defined by the ≤5% and ≤1% thresholds. The histogram in Figure 7 breaks down the distribution in greater detail.

### Qualitative Analysis

Because the arcuate model, in part, will be used to visually aid clinicians in their interpretation of the glaucoma report, we wanted to perform a qualitative assessment of the arcuate model trajectories on the RNFL p-map.

#### RNFL p-Map

First and foremost, the arcuate model predicts retinal nerve fiber bundle trajectories. As such, the model's fit to the RNFL p-map is most important. We selected an example from the superior retina and inferior retina to illustrate our observations.

**Figure 8.**
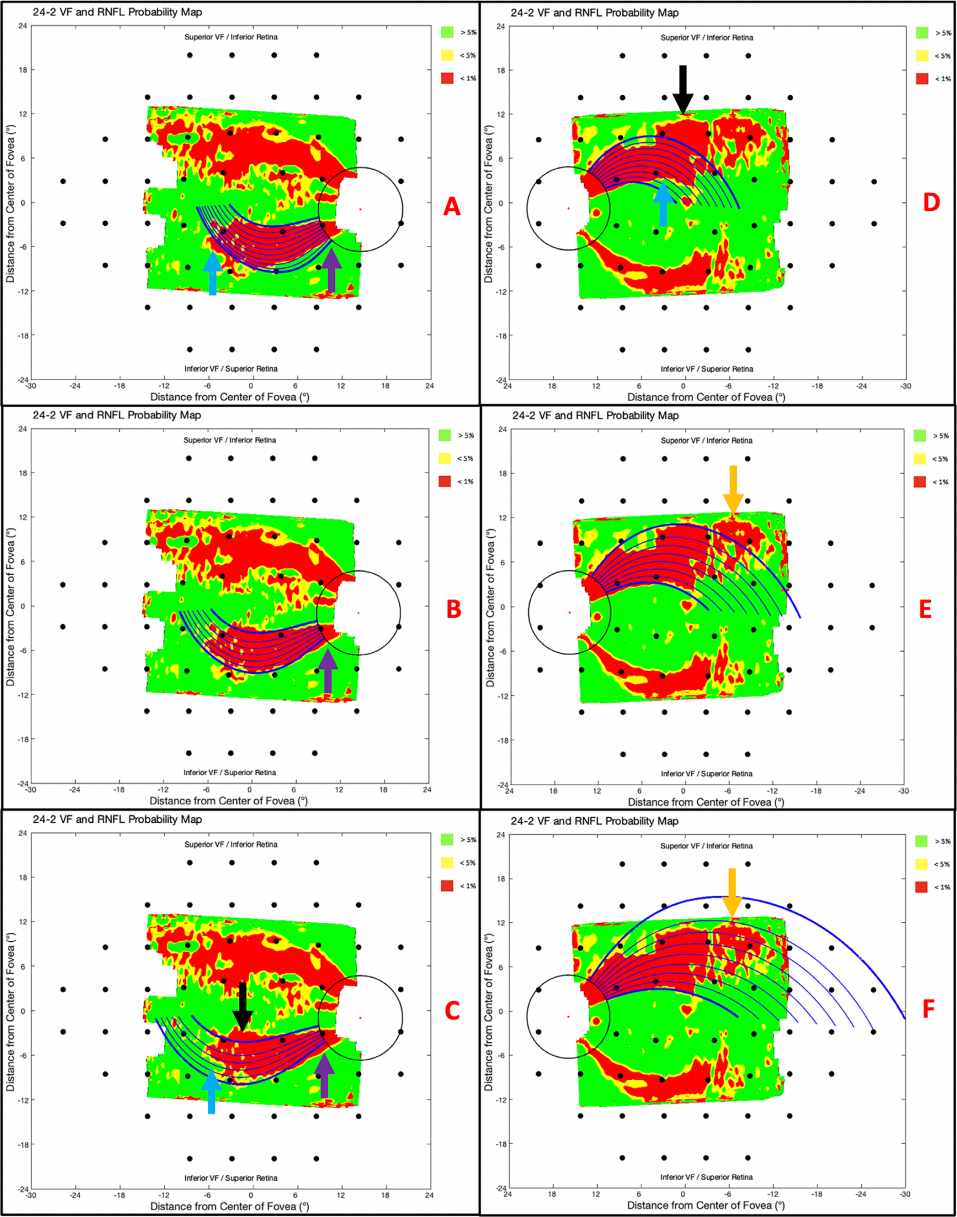
The best-fit (degree position) trajectories for β_Sup, Max_ = −1.3 (A), β_Sup, Mean_ = −1.9 (B), β_Sup, Max_ = −2.5 (C), β_Inf, Max_ = 1.3 (D), β_Inf, Mean_ = 0.7 (E), and β_Inf, Min_ = 0.1 (F) plotted on the RNFL p-maps with superior border issues (*blue arrows*), inferior border issues (*black arrow*), disc issues (*purple arrows*), and an irregularity (*orange arrow*) marked.

Figures 8A–C demonstrate the best-fit degree positions for an arcuate pattern in the superior retina via the parameters β_Sup, Max_ = −1.3 (Fig. 8A), β_Sup, Mean_ = −1.9 (Fig. 8B), and β_Sup, Min_ = −2.5 (Fig. 8C). None of the β_Sup_ parameters produce trajectories which match the superior arcuate pattern's borders perfectly. In this case, the β_Sup, Max_ trajectories clearly miss the superior edge of the arcuate pattern (Fig. 8A; blue arrow) and struggle to align near the disc (Fig. 8A; purple arrow). The β_Sup, Min_ trajectories miss both the superior edge (Fig. 8C; blue arrow) and inferior edge (Fig. 8C; black arrow) of the arcuate pattern and, similarly, struggle to align near the disc (Fig. 8C; purple arrow). The β_Sup, Mean_ trajectories struggle to align near the disc, but otherwise align very well with the arcuate pattern. Overall, the mean β_Sup_ value appeared to fit reasonably well to the borders of the arcuate pattern.

Figures 8D–F demonstrate the best-fit degree positions for an arcuate pattern in the inferior retina via the parameters β_Inf, Max_ = 1.3 (Fig. 8D), β_Inf, Mean_ = 0.7 (Fig. 8E), and β_Inf, Max_ = 0.1 (Fig. 8F). None of the β_Inf_ parameters produce trajectories that match the inferior arcuate pattern's borders perfectly. For this eye, the β_Inf, Max_ trajectories clearly miss both the inferior edge (Fig. 8D; black arrow) and superior edge (Fig. 8D; blue arrow) of the arcuate pattern. The β_Inf, Min_ trajectories seem to align with an indeterminate portion of the arcuate pattern in the periphery (Fig. 8F; orange arrow). The β_Inf, Mean_ trajectories, on the other hand, miss the indeterminate portion of the arcuate pattern in the periphery (Fig. 8E; orange arrow). It should be noted that the indeterminate portion of the arcuate pattern may be the result of ≤1% thresholding; in other words, this portion of the pattern may belong to more peripheral retinal nerve fiber bundles that either failed the threshold or were outside the field-of-view of the scan. In any case, the mean β_Inf_ value appeared to fit reasonably well to the borders of the arcuate pattern.

With few exceptions, the mean β parameters produced arcuate lines that corresponded well to the trajectory of the arcuate-shaped patterns.

## Discussion

We used eyes with glaucomatous optic neuropathy, which are characterized by their arcuate-like pattern of thinning of the retinal nerve fibers, to perform a test of our modified version of the Jansonius et al. model.^9^^,^^10^ The arcuate model's predicted retinal nerve fiber bundle trajectories closely aligned with the borders of the arcuate-shaped patterns in the OCT RNFL p-maps. Further, the mean β_Sup_ and β_Inf_ parameters rarely yielded trajectories that differed significantly, relative to the other β values, from the arcuate borders. Thus, for clinical use, adjusting for individual differences in best-fitting β values is rarely, if ever, needed.

**Figure 9.**
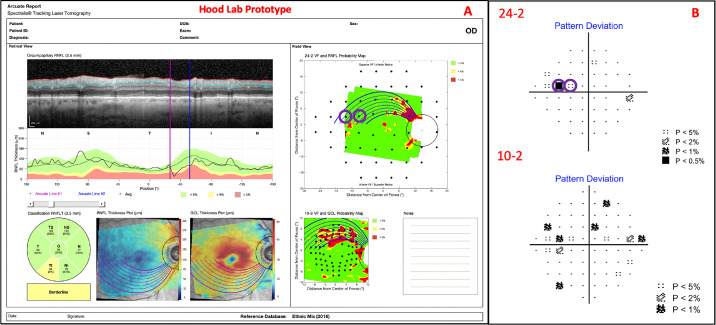
An arcuate report enhances aS-aF agreement for a glaucomatous eye (A) and 24-2 and 10-2 visual fields (B).

In terms of our larger goals, adding arcuate trajectories to an OCT report, as in Figure 5, should make it easier, in the future, to compare abnormal regions on the RNFL p-map to those on 24-2 VFs, especially when the abnormal 24-2 VF locations are outside the RNFL p-map. This is of particular interest, as others have documented the potential of structure-function agreement/indices in the detection of early glaucoma.^15^^–^^19^ Figure 9 presents an example of an eye with a clear arcuate-shaped pattern. Interestingly, an abnormal structure-abnormal function (aS-aF) quantitative metric employing the arcuate model would have achieved enhanced aS-aF agreement (Fig. 9). The GCL p-map and 10-2 VF have good aS-aF agreement, but the RNFL p-map and 24-2 VF do not. The purple circles demonstrate the abnormal structure (Fig. 9A; purple circles) and abnormal function (Fig. 9B; purple circles) agreement that would be achieved by extending the abnormal region's trajectories. In other words, the trajectories would increase our sensitivity and decrease the number of false negatives for our laboratory's automated structure-function agreement method.^7^ Similar adjustments to structure-function mapping,^20^^,^^21^ based on the model by Jansonius et al., have previously shown improved concordance in some eyes.

**Figure 10.**
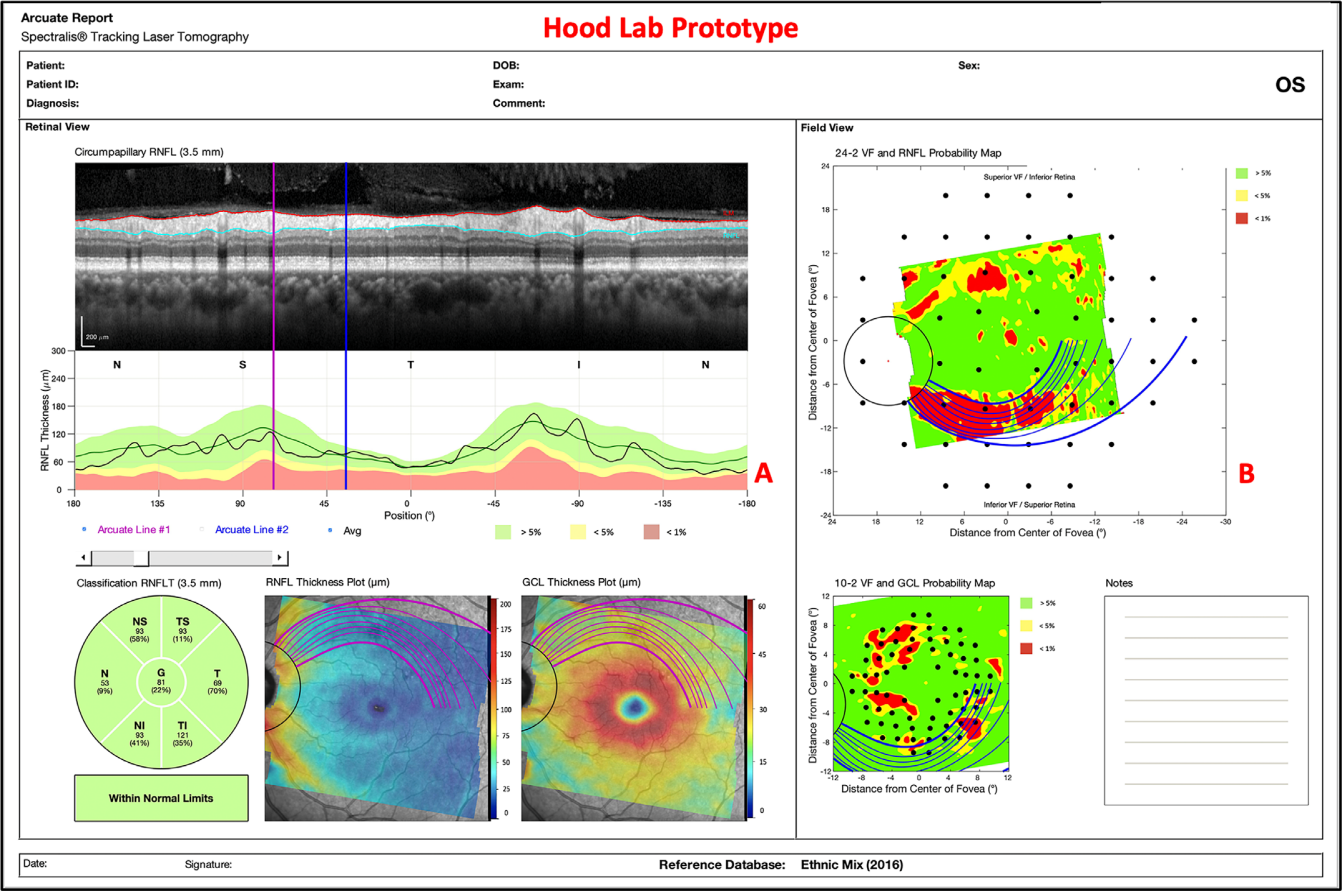
An example of subtle damage on the cpRNFL plot (A), but clear arcuate-shaped pattern on the RNFL p-map (B).

The arcuate report will also help the clinician to better understand the relationship between the cpRNFL plot and the RNFL p-map. These are both commonly included in OCT reports. Thus it is important to understand how they are related. The arcuate model can help in two ways. First, the model indicates the corresponding regions on the cpRNFL plot and RNFL p-map. In many cases, the corresponding region on the cpRNFL plot will fall into the red regions and a clear thinning will be apparent on the b-scan image, as seen in Figure 5. However, not every arcuate-shaped pattern yields an abnormal region on the cpRNFL plot. [Note that on average, only 68% and 37% of the area predicted by the trajectory-determined regions fell below the ≤5% and ≤1% cpRNFL thresholds, respectively.] This is important, particularly if clinicians favor the older cpRNFL plot over the relatively newer RNFL p-map as they may miss clear glaucomatous damage, such as that shown on the RNFL and GCL p-maps in Figure 10.

**Figure 11.**
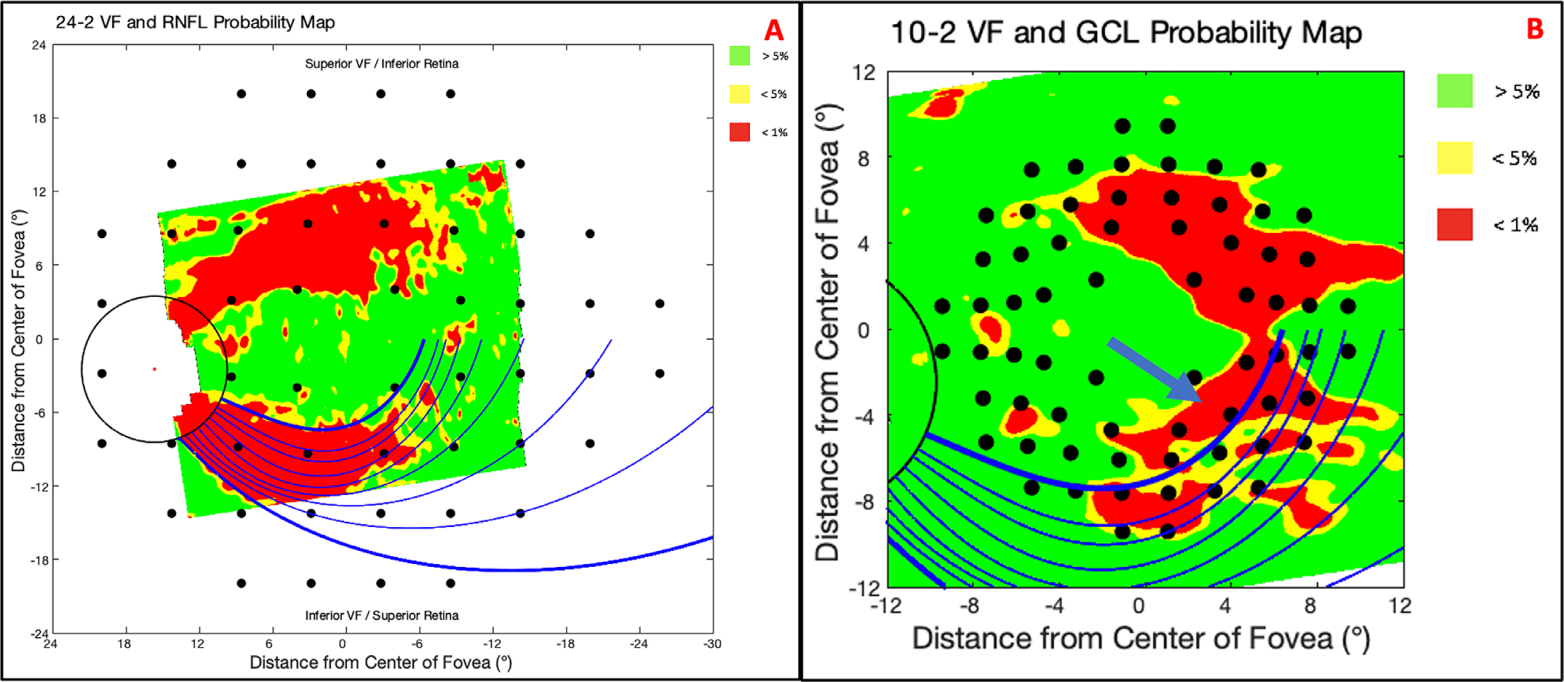
The best-fit (degree position) trajectories for mean β_Sup_ on the RNFL p-map (A) and GCL p-map (B).

On a similar note, the inclusion of the arcuate trajectories on the GCL p-map should help clinicians gain a better understanding of the GCL damage associated with glaucomatous arcuate patterns. We,^22^ and others,^23^^,^^24^ have highlighted the importance of the macular region and the high frequency of macular RGC loss even in eyes with early glaucomatous damage. Therefore it is imperative to improve a clinician's ability to detect topographically consistent damage between the RNFL and the GCL. Using the mean β_sup_ and β_Inf_ parameters, we extended best-fitting trajectories for the RNFL p-maps (Fig. 11A) to the GCL p-maps to qualitatively assess the fit to the abnormal GCL regions. In 31 of the 31 eyes, the abnormal regions of the GCL p-map fell primarily within the predicted regions of the model. However, in 29 of the 31 eyes, the abnormal region of the GCL p-map was skewed toward the area between the model's predictions and fixation (Fig. 11B; blue arrow). This is not surprising because the model was not designed for retinal ganglion cells. Additionally, as Hood^5^ points out, the GCL is thickest near fixation, while the RNFL is thickness near the disc, and the abnormal regions are going to be more prominent in the thicker regions.

Additionally, it should be noted that the arcuate trajectories on the GCL p-map may play a critical role in an OCT-based definition of glaucoma. In particular, we^8^^,^^25^ have recently shown that abnormal regions on the RNFL and GCL p-maps that are part of the same arcuate region are a sufficient condition for identifying optic neuropathy consistent with glaucoma. Adding the trajectories from the model to reports as in Figures 5, 9, and 10 should aid in the qualitative and quantitative evaluations of RNFL and GCL p-maps.

### Limitations

The sample size was relatively small as it is difficult to find glaucomatous eyes with clear superior arcuate-shaped patterns without other regions affected. Additionally, although it was difficult to find a better alternative, the somewhat arbitrary ≤1% thresholds for the arcuate pattern may have led to some mischaracterization of the true arcuate border. Finally, it is unfortunate that more customized β parameters for an individual eye would necessitate acquiring additional information on spherical equivalent refraction, axial length, optic disc anatomy, or retinal blood vessel topography.^10^^,^^12^^,^^13^ This is not practical in a clinical setting and, even with this information, it can only partially account for location-specific intersubject variability.

Care is also needed to avoid fitting the model to arcuate-like artifacts seen on the RNFL p-maps of healthy eyes. In a recent study of 200 healthy control eyes, 4% of the RNFL p-maps had arcuate-like abnormal regions. However, Hood et al.^14^ illustrated that these arcuate patterns could be identified as artifacts, as opposed to glaucomatous arcuates, if they met both of the following criteria: (1) they do not cross the vertical meridian because they will be constrained to the thick region of RNFL in the average RNFL thickness map and (2) they do not have a topographic corresponding abnormal region on the GCL p-map. Note that most of the RNFL p-maps showed an arcuate region that crossed the meridian, and all had an abnormal region on the GCL p-map that topographically corresponded.

## Summary

In summary, a modified version of the model by Jansonius et al. can predict the trajectories of the retinal nerve fiber bundles in eyes with glaucomatous optic neuropathy. Adding the results of this model to OCT reports should help clinicians see the topographical relationship between abnormal regions on different aspects of the OCT report, including the cpRNFL b-scan, cpRNFL plot, RNFL thickness plot, GCL thickness plot, RNFL p-map, and GCL p-map. It should also be helpful in performing structure-function agreement analysis in the region of the retina outside the OCT scan. Ultimately, the model's trajectories should increase a clinician's confidence in a diagnosis of glaucomatous optic neuropathy if and only if it improves structure-structure or structure-function agreement.
